# Tibial Eminence Avulsion Fracture in Pediatric Patients Reinserted with Arthroscopy and Pull-Out Suture Technique: Clinical and Functional Outcomes in a Long-Term Follow Up with Return to Sport

**DOI:** 10.3390/children12040499

**Published:** 2025-04-14

**Authors:** Franziska M. Kocher, Ludovic Galofaro, Joseph M. Schwab, Ines Raabe, Moritz Tannast, Daniel Petek

**Affiliations:** 1 Unit of Orthopedics and Traumatology, HFR-Fribourg, 1708 Fribourg, Switzerland; ines.raabe@h-fr.ch (I.R.);; 2 Unit of Emergency Medicine, HFR-Fribourg, 1708 Fribourg, Switzerland; 3 Section of Medicine, University of Fribourg, 1700 Fribourg, Switzerland; joseph.schwab@unifr.ch; 4 Unit of Orthopedics and Traumatology, Inselspital Bern, 3010 Bern, Switzerland; moritz.tannast@insel.ch

**Keywords:** TEAF, ACL, pediatric population, PROMs, Pedi-IKDC, Lysholm, Tegner, Marx, return to sport

## Abstract

**Background/Objective:** Tibial eminence avulsion fracture (TEAF) is a traumatic injury of the anterior cruciate ligament that occurs in children with an immature skeletal system. It has an incidence of 3 per 100,000 children, with an increasing prevalence over time. The objective of this study was to evaluate the long-term clinical and subjective outcomes of displaced TEAF requiring surgical intervention and to assess the return to sport. **Methods:** A retrospective cohort study was conducted, identifying all patients who underwent arthroscopic-assisted TEAF refixation at HFR-Fribourg between 2012 and 2020, performed by a single surgeon. A total of fifteen patients were included for descriptive analysis, while twelve patients underwent clinical assessment of knee joint stability and functional outcomes using patient-reported outcome measures (PROMs): Pedi-IKDC, Lysholm, Tegner, and Marx. **Results:** Of the fifteen patients, fourteen had type III and one had type II TEAF according to the McKeever classification. The mean age at the time of injury was 11.5 years, and the average time between surgery and long-term follow-up was 5.1 years (range: 0.9–8.9 years). For the primary outcomes of the operated knee, no significant differences were observed in muscle mass or range of motion between the operated and non-operated limbs (*p* > 0.05). Anterior knee stability, as assessed by the Lachman test and Rolimeter, showed no significant difference between the operated and non-operated knee (*p* > 0.05). Regarding secondary outcomes, the Pedi-IKDC and Lysholm scores were 98 out of 100, the Tegner score was 5.5 out of 10, and the Marx score was 14.5 out of 20 at the final follow-up. There were no significant differences in the number of hours per week or frequency of sport activity (mean three times per week) before and after surgery. **Conclusions:** The surgical treatment for displaced TEAF, specifically the pull-out suture technique with arthroscopic assistance, demonstrates excellent clinical and functional outcomes, with high recovery rates and restored knee stability. This technique allows patients to return to sports without significant impairment. Although no comparative analysis was performed, these findings provide a foundation for future studies to further validate and compare the effectiveness of this surgical approach.

## 1. Introduction

### 1.1. Background and Rationale

**Figure 1:**
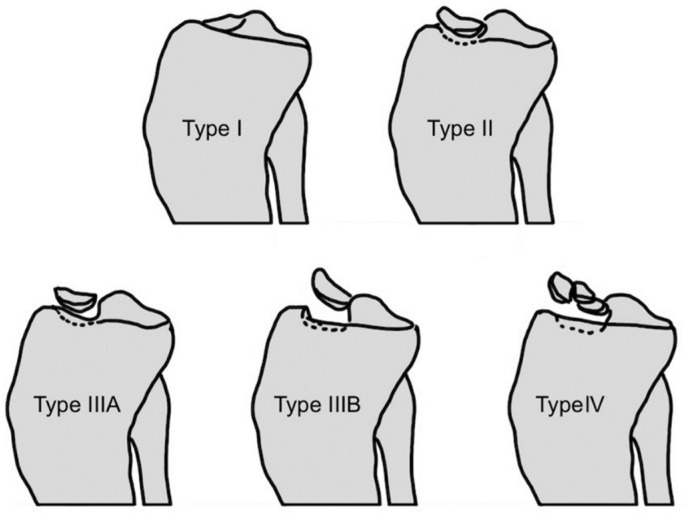
TEAF classification according to Meyers and McKeever modified by Zaricznyj.

Tibial eminence avulsion fractures (TEAF) are relatively rare injuries, predominantly affecting pediatric patients between the ages of 7 and 18. This type of fracture is most often seen in children who participate in sports that involve high kinetic energy. As athletic activities in this age group have transitioned from multiple sports to increased single-sport intensity and training, there has been an increasing incidence of these injuries [1]. The occurrence of TEAF is relatively low, with a reported rate of 2.8 cases per 100,000 individuals per year [2]. These fractures typically result from mechanisms such as knee hyperextension, flexion combined with valgus, and internal rotation of the tibia [3]. The classification of TEAF, established by Meyers and McKeever and modified by Zaricznyj, divides fractures into four types based on the displacement and position of the avulsed tibial spine fragment (Figure 1) [4,5]. Surgical treatment options for displaced fractures (types III and IV) generally include screw fixation or suture fixation after reduction [6].

Despite clinical–radiological diagnosis and management strategies for TEAF being well established, there is limited research on the long-term subjective and functional outcomes, particularly in terms of the patient’s ability to return to sport. Existing studies predominantly focus on clinical outcomes such as fracture healing and knee stability but fail to address the functional implications for young athletes who wish to resume their sports activities [7]. The lack of long-term follow-up data on the return-to-sport rates, as well as the effectiveness of various surgical approaches in achieving this goal, presents a significant gap in the literature [8].

### 1.2. Aims

We therefore asked the following questions:

(1) What is the long-term clinical function in terms of the patient’s ability to return to sport and the objective anterior stability of the knee joint, as evaluated by Lachman test and Rolimeter, compared to the non-operated knee after arthroscopic surgical refixation for displaced type II and III TEAF?

(2) What are the long-term patient-reported outcomes (PROMs), including Pedi-IKDC, Lysholm, Tegner, and Marx scores, for patients with displaced TEAF treated with surgical refixation, and how do these scores correlate with return-to-sport success?

(3) How does the frequency and duration of sport participation change pre- and post-operatively for pediatric patients with displaced TEAF?

(4) What is the overall re-operation rate for included patients who underwent surgical refixation?

## 2. Materials and Methods

### 2.1. Study Design

This is a retrospective single-center, observational cohort study, evidence class IV. The observational clinical study protocol (2022-01657) was accepted by the Ethics Committee of Canton de Vaud, Switzerland, on the 1 June 2023. This study involves 15 patients who underwent arthroscopic knee surgery at the HFR-Fribourg, Switzerland, between 1 January 2012 and 31 December 2020 by the same surgeon, with an identical surgical treatment for refixation of the avulsed tibial spine.

### 2.2. Participants and Setting

Pre-screening of patients corresponding to the eligibility criteria (Table 1) was performed from the HFR-Fribourg’s internal server. We selected patients who had been operated on in a timespan of eight years. Each patient had undergone the same operative technique by the same surgeon. Data from the follow-ups were collected. A routine follow-up was scheduled six weeks post-operatively. The long-term follow-up was scheduled after a period of at least six months post-operatively.

### 2.3. Source Data and Quantitative Variables

For the collection of source data, fracture classification, assessment of preoperative status, imaging, and postoperative data (after 6 weeks), the HFR-Fribourg server was used. For data collected during the long-term follow-up, all patients were contacted for clinical examination and completion of questionnaires.

The primary variables of interest were the Lachman test, the anterior drawer measured by Rolimeter [9], the pivot shift [10], the range of motion (ROM) [11], and muscle atrophy. The Lachman test was described as “firm stop”, “smooth stop”, or “no stop”, and the residual laxity was measured in cm. An ACL deficiency was defined as a difference of 3 mm or more compared to the non-operated knee [9].

Validated questionnaires were used to asses patient-reported outcomes (PROMs): Pedi-IKDC [12,13]. Lysholm score was chosen to assess subjective knee instability and the Tegner and Marx scores to assess sports practice [14].

### 2.4. Description of Used Surgical Pull-Out Suture Technique

**Figure 2:**
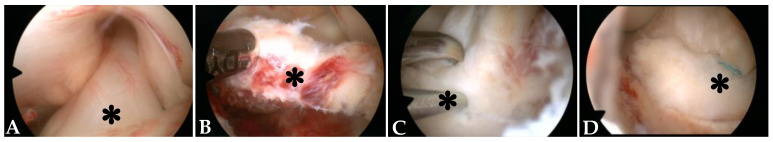
Intraoperative arthroscopic images: (**A**) front view of a pediatric ACL (*); (**B**) bottom view of the bony avulsion of ACL insertion (*); (**C**) reduced avulsion and fixation with percutaneous K-wire (*); (**D**) control of the reduced TEAF thanks to the pull-out suture technique and the Ti-Cron 5TM in green (*).

Under general anesthesia, with the patient in a supine position and a tourniquet applied, standard anteromedial and anterolateral arthroscopic portals are established to provide access to the ACL (Figure 2A). To enhance the visualization and exposition of the TEAF, the synovial membrane, infrapatellar fatty tissue (Hoffa’s fat pad), and hematoma surrounding the fracture site are partially resected (Figure 2B). Interposition of the meniscus and transverse ligament are frequently observed, as noted by Kocher et al. [15]. These structures are retracted using a bone hook to facilitate reduction of the TEAF. The avulsion is reduced and temporarily fixed with a percutaneous K-wire (Figure 2C).

**Figure 3:**
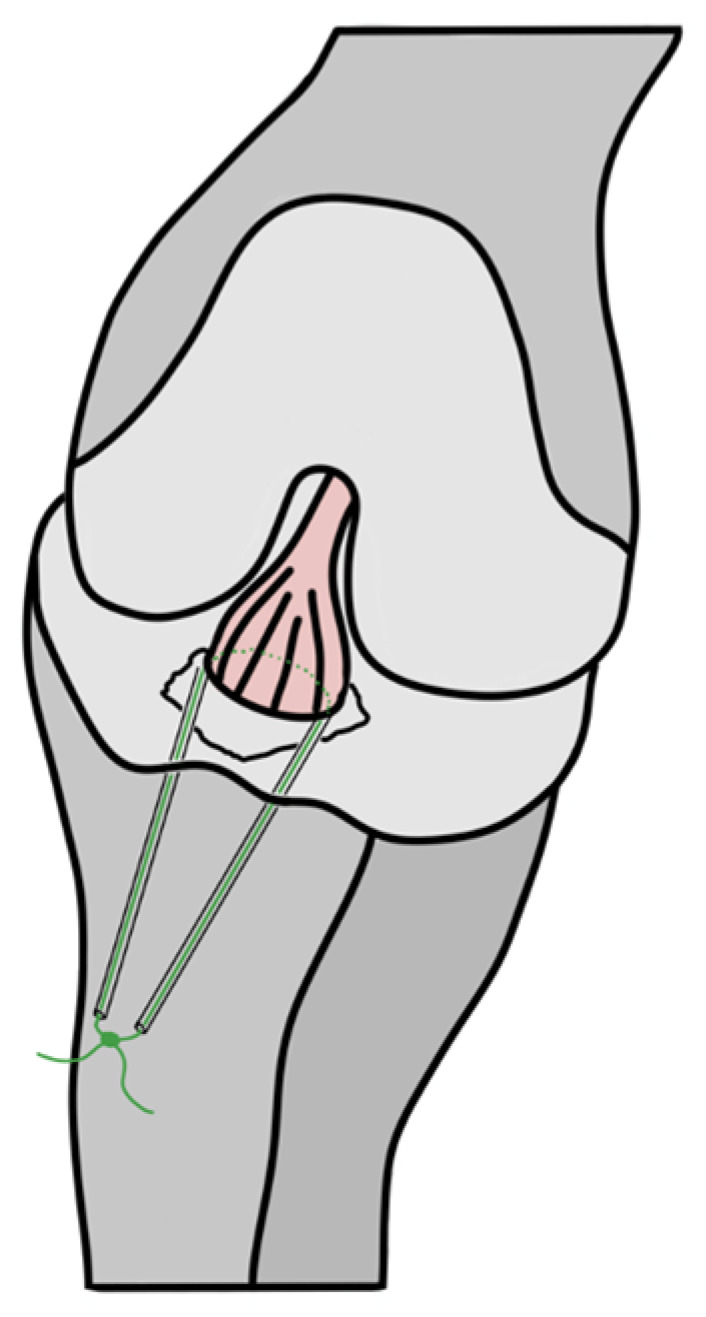
Refixation of a TEAF using a pull-out suture fixation technique (adapted image from Tercier et al. [1], used with permission).

A Ti-Cron 5™ suture (Braided Polyester Sutures, Medtronic, Minneapolis, MN, USA) is passed through the bony avulsion of the ACL. Two tunnels are then drilled using guides from a meniscal root repair system, placed adjacent to the medial and lateral edges of the avulsed fragment’s base. The suture is passed through these tunnels, pulled tight, and secured to the anterior proximal tibial surface to reduce the fracture and stabilize the TEAF (Figure 3). The suture is tightened with the knee positioned in slight flexion (approximately 10°). Final fracture reduction is assessed arthroscopically (Figure 2D), and joint stability is tested during flexion-extension movements [16].

### 2.5. Statistical Method

Continuous variables were reported as means with standard deviations. Categorical variables were expressed as absolute frequencies and percentages, with associations evaluated via the Chi-square test. Paired samples were compared using Student’s t-test. The normality of the continuous data was verified with the Shapiro–Wilk test, and homogeneity of variance was assessed using Bartlett’s test. Data analysis was performed using RStudio version 1.4.1106 and Microsoft Excel. The statistical significance threshold was set at α < 0.05, with a 95% confidence interval.

For descriptive statistics, all available data were presented, acknowledging any variations in the number of observations. A descriptive analysis was performed to evaluate gender differences in prevalence, comparing boys and girls. Results were presented as absolute frequencies or percentages. Missing data were excluded from the statistical analyses and reported in the results. Given the small sample size, sensitivity analyses were not feasible.

## 3. Results

### 3.1. Participants

Between 2012 and 2020, eighteen patients were operated on for displaced TEAF by one surgeon. Three patients were excluded from the study because they did not meet the eligibility criteria. The reasons were the presence of an associated fracture of the tibial plateau, a different surgical technique with screw fixation, and the presence of a spastic neuromuscular disease. Of the fifteen patients who met the selection criteria, three could not be included: one could not be contacted and the other two refused to participate for follow-up in the long-term study. Nevertheless, their data were used for the descriptive results.

### 3.2. Descriptive Data

Table 2 presents the descriptive data for the 15 included patients. Gender distribution was 66% boys and 34% girls, with no significant gender difference (*p* = 0.682). The mean age at the time of injury was 11.5 ± 0.6 years. TEAFs occurred during sports activities in 73.3% of cases: skiing (eight cases), running (three cases), cycling, football, gym, and motorcycling (one case each). Fracture types included 14 type III fractures (93.33%) and 1 type II fracture (6.67%). All patients had pre- and post-operative standard radiographs. Seven patients had preoperative MRIs and five had CT scans.

### 3.3. Primary Outcomes

The mean follow-up time for the 12 patients included in the long-term follow-up was 5.1 ± 0.2 years, ranging from 0.9 to 8.9 years. The mean BMI at follow-up was 22.55 kg/m^2^. Anterior knee stability parameters, as shown in Table 3, were compared between the operated and non-operated knee of the patient.

**Figure 4:**
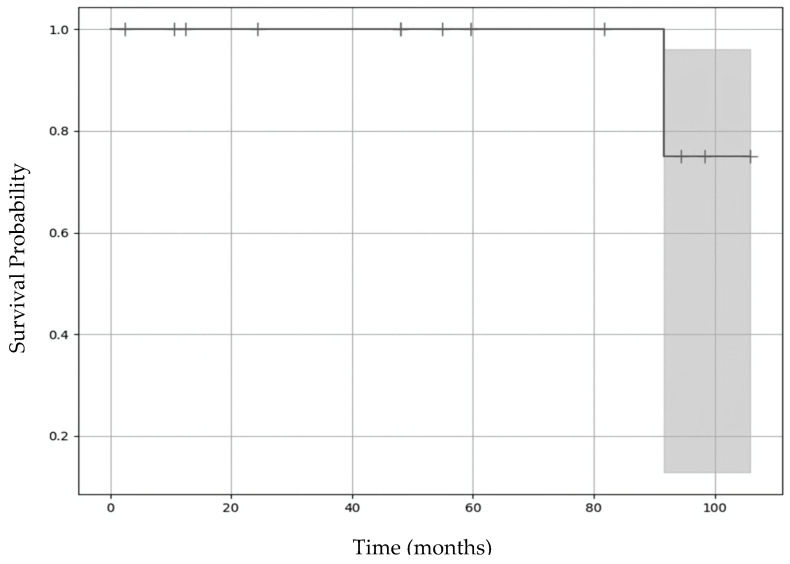
Kaplan–Meier survival curve for the operated knee (excluding missing long-term follow-ups). The shaded area represents the confidence interval.

The Kaplan–Meier survival curve for the operated knee showed a high probability of avoiding re-operation throughout the follow-up period, with a slight decrease at the end, reflecting one re-operation. The shaded area represents the confidence interval (Figure 4).

Muscle mass, range of motion, and anterior knee stability were measured, and the results are presented in Table 3 for the 12 patients. The thigh circumference was 43.6 ± 2.1 cm for the operated knee and 43.8 ± 2.0 cm for the non-operated knee (*p* = 0.57). The calf circumference was 33.0 ± 2.1 cm for the operated knee and 33.5 ± 2.1 cm for the non-operated knee (*p* = 0.19). Knee flexion was 137.7 ± 3° for the operated knee and 139.1 ± 3° for the non-operated knee (*p* = 0.34), while knee extension was 4.6 ± 1.3° for the operated knee and 4.8 ± 1.2° for the non-operated knee (*p* = 0.90). For knee stability, the Lachman test showed no translation for the operated knee and 0.2 ± 0.2 cm for the non-operated knee (*p* = 0.34). The Rolimeter test showed 5.4 ± 0.5 mm for the operated knee and 5.4 ± 0.7 mm for the non-operated knee (*p* = 1.0). The pivot shift test for all patients was negative.

### 3.4. Secondary Outcomes

The secondary objectives are shown in Table 4. The Pedi-IKDC score was 98 out of 100, the Lysholm score was 98.5 ± 0.32 out of 100, the Marx score was 14.0 ± 0.25 out of 16, and the Tegner score was 5.42 ± 0.14, indicating a high level of sport activity and the ability to return to a competitive level. No severe complications were reported. Regarding return to sport, the comparison between pre-operative and final follow-up data, expressed in terms of time, with a mean activity of 3 h/week, and frequency, with a mean of three sessions/week.

## 4. Discussion

Tibial eminence avulsion fractures (TEAF) are rare injuries that occur primarily in pediatric patients and are often associated with high-energy sports activities. Surgical treatment is recommended for displaced fractures by using, for example, an arthroscopic controlled pull-out suture technique. While much of the existing literature on TEAF has focused on the clinical and radiological management of the injury, there is a lack of research addressing the long-term subjective outcomes and return to sport. This study aims to fill that gap by assessing the knee joint stability, functional outcomes, and return-to-sport rates in patients treated for displaced TEAF using a standardized surgical technique. This study found that surgical refixation of displaced TEAF results in excellent long-term functional outcomes, with most patients able to return to their pre-injury level of sport activity. The lack of significant differences between the operated and non-operated knees in terms of stability and range of motion suggests that the surgical technique used does not impair their knee function. Clinicians should consider these findings when advising patients and their families on the expected outcomes following surgery, as well as when planning post-operative rehabilitation aimed at maximizing return-to-sport success.

### 4.1. Long-Term Function and Objective Anterior Stability of the Knee Joint

The main finding of our study is that the arthroscopic-assisted surgical technique with a pull-out suture used for treating displaced type II and III TEAF resulted in excellent long-term knee stability, with no significant differences between the operated and non-operated knees in terms of anterior translation, range of motion, or muscle mass. This is important because it indicates that the surgical procedure not only restores knee stability but also maintains functional outcomes, allowing patients to return to sports without compromising their knee function. Surgeons can confidently recommend this technique for managing TEAFs, knowing it restores knee stability and enables recovery comparable to the uninjured knee.

Our findings with comparable anterior knee stability using the Lachman test and the quantifying anterior drawer test by using the Rolimeter are consistent with the literature, such as studies by Gans et al. [17] and Coyle et al. [18], which reported high knee stability after surgery. However, in contrast to some studies that observed residual anterior laxity [19], our results suggest a more consistent restoration of stability. This may be attributed to the standardized surgical protocol used in our study and an experimented single surgeon, which appears to have minimized the risk of residual laxity. These findings emphasize the importance of a consistent and well-established standardized surgical technique and setting for achieving optimal long-term stability.

In comparison to other studies that reported complications such as arthrofibrosis, non-union, or malunion, our study found no cases of these issues, suggesting that the surgical approach we employed is effective in avoiding possible complications. Specifically, we observed no loss of range of motion or muscle atrophy in the long term, aligning with findings from Casalonga et al. [19], who reported that joint mobility was preserved in pediatric patients after similar surgeries. Future studies should also focus on refining rehabilitation protocols to minimize complications like arthrofibrosis and ensure optimal recovery for all patients.

### 4.2. Long-Term Functional Subjective Outcomes of Surgically Treated Patients with Displaced TEAF in Terms of Patient-Reported Outcomes (PROMs) and Return to Sport

Our study found that most patients had excellent long-term (mean 5 years follow-up) functional outcomes, with high scores on the Pedi-IKDC (98 of 100), Lysholm 98.5 of 100), and Tegner scales, and 95% returned to sport at their pre-injury levels. This highlights that arthroscopic surgery for displaced type II and III TEAFs can enable patients, particularly adolescents, to resume normal physical activities with minimal restrictions. Surgeons can confidently inform patients and families that with proper rehabilitation, a full return to sport is achievable.

Our findings align with previous studies, such as those by Gans et al. and Coyle et al., which also report high functional scores after arthroscopic surgery [17,18]. However, unlike other studies, our cohort showed a 95% return to sport at the same frequency and intensity as the pre-injury level, underscoring the effectiveness of this approach. In contrast, Casalonga et al. [19] found that only half of their patients returned to sport at the same level, which could be due to different patient populations or rehabilitation protocols [4]. Stallone et al. also reported a 23% drop in sports participation, attributing it to psychological factors such as fear of re-injury [8].

Despite the high return to sport, some studies, including ours, report patients with mild residual laxity who still perform well functionally [3,20]. This suggests that subjective outcomes, like PROMs, can sometimes be decoupled from objective findings. Future research should examine the long-term effects of residual laxity and its potential link to osteoarthritis or secondary injuries.

### 4.3. Reoperation

Our study identified one patient who required ACL reconstruction eight years post-operatively following a skiing accident. This is consistent with findings from Stallone et al. [8], who reported similar low reoperation rates. This highlights the potential for future ACL injuries, particularly in younger patients, which should be communicated to all patients undergoing TEAF surgery. Future studies should explore the long-term risk of ACL tears in this population to better understand the relationship between TEAF and subsequent knee injuries.

### 4.4. Perspectives and Limitations

This study has several limitations. First, the small sample size (n = 12) and monocentric design limit generalizability. A larger, multicentric study would provide more robust data. Additionally, the retrospective design introduces potential recall bias, as patient-reported outcomes and clinical parameters were assessed systematically only at the latest follow-up. A medium-term follow-up with repeated PROMs would offer a better understanding of recovery progression.

The literature on TEAF primarily consists of small, retrospective studies, which limits generalizability and is prone to bias. A multicentric study is needed to establish clear guidelines on TEAF management. Moreover, while surgical techniques were clinically evaluated, further biomechanical studies are required to compare these different techniques in terms of fixation stability and anterior knee stability in the long term. Lastly, the retrospective evaluation of return to sport at 6 months is controversial as young patients often change activities for reasons unrelated to their knee, and there are also psychological factors influencing sports participation after ACL refixation, e. g., fear or apprehension.

## 5. Conclusions

This study demonstrates that surgical treatment for displaced TEAF, using a standardized arthroscopic pull-out suture technique, results in excellent long-term functional outcomes and anterior knee stability, with most patients returning to sports at their pre-injury levels. Future studies should focus on larger, multicenter cohorts to validate these findings and explore biomechanical differences between surgical techniques. Additionally, examining the long-term risks of residual laxity, the relationship between TEAF and subsequent ACL injuries, and the psychological factors affecting recovery would further refine treatment approaches and improve patient outcomes.

## Figures and Tables

**Table 1:** Eligibility criteria.

Inclusion Criteria	Exclusion Criteria
<16 years of age at the time of the accident	Not signed ICF or unwilling to participate
Open growth line with displaced TEAF	Multiple ligament or cartilage damages
TEAF type II to IV requiring surgical treatment	TEAF type I or II with conservative treatment
Pre- and post-radiological images	Fracture of the tibial plateau with osteosynthesis
Identical surgical technique: reduction with arthroscopy and fixation with pull-out suture	Other surgical technique or fixation method: arthrotomy or fixation with pins or screws
	Neuromuscular disease
	Intra-substantial tear of the ACL

**Table 2:** Patient demographics in percentage of included patients (n = 15).

Parameter	Value
Sex	
Male	60
Female	40
Side	
Right	40
Left	60
Fracture type according to Meyer–McKeever	
II	6.67
III	93.33
Pre-operative additional imaging to radiographs	
MRI	46.67
CT scan	33.33
No	20
Injury mechanisme	
Ski	53.33
Running	20
Football	6.67
Bike	6.67
Gym	6.67
Motorbike	6.67

**Table 3:** Muscle mass, range of motion, and knee stability at long-term follow-up. Values of continuous parameters as mean ± SD.

Parameter	Operated Knee	Non-Operated Knee	*p*-Value
Thigh circumference (cm)	43.6 ± 2.1	43.8 ± 2	0.57
Calf circumference (cm)	33 ± 2.1	33.5 ± 2.1	0.19
Knee flexion (degrees)	137.7 ± 3	139.1 ± 3	0.34
Knee extension (degrees)	4.6 ± 1.3	4.8 ± 1.2	0.90
Lachman (cm)	0 ± 0	0.2 ± 0.2	0.34
Rolimeter (mm)	5.4 ± 0.5	5.4 ± 0.7	1

**Table 4:** Sport activity at long-term follow-up and before operation. Values of continuous parameters as mean ± SD.

Sport Activity	Last Follow-Up	Pre-Operative	*p*-Value
Hours/week	2.92 ± 0.08	3.0 ± 0.08	0.76
No. of sessions/week	3.08 ± 0.15	3.25 ± 0.12	0.62

## Data Availability

Data are unavailable due to privacy or ethical restrictions. The data presented in this study are available on request from the corresponding author.
